# Stroop effects from newly learned color words: effects of memory consolidation and episodic context

**DOI:** 10.3389/fpsyg.2015.00278

**Published:** 2015-03-12

**Authors:** Sebastian Geukes, M. Gareth Gaskell, Pienie Zwitserlood

**Affiliations:** ^1^Institut für Psychologie, Westfälische Wilhelms-Universität MünsterMünster, Germany; ^2^Department of Psychology, University of YorkYork, UK

**Keywords:** Stroop effect, novel-word learning, semantic learning, memory consolidation, complementary learning systems, episodic context, color matching

## Abstract

The Stroop task is an excellent tool to test whether reading a word automatically activates its associated meaning, and it has been widely used in mono- and bilingual contexts. Despite of its ubiquity, the task has not yet been employed to test the automaticity of recently established word-concept links in novel-word-learning studies, under strict experimental control of learning and testing conditions. In three experiments, we thus paired novel words with native language (German) color words via lexical association and subsequently tested these words in a manual version of the Stroop task. Two crucial findings emerged: When novel word Stroop trials appeared intermixed among native-word trials, the novel-word Stroop effect was observed immediately after the learning phase. If no native color words were present in a Stroop block, the novel-word Stroop effect only emerged 24 h later. These results suggest that the automatic availability of a novel word's meaning depends either on supportive context from the learning episode and/or on sufficient time for memory consolidation. We discuss how these results can be reconciled with the complementary learning systems account of word learning.

## Introduction

Learning a foreign language after childhood entails the acquisition of the rules of grammar of the novel language, knowledge that may arguably become explicit with practice, as well as learning a tremendous number of new words, as labels for concepts that have been acquired and mapped onto native words during first-language acquisition. Words as labels for concepts constitute explicit knowledge, and in the course of learning a new language, the human mental lexicon, which stores word knowledge, may double in size. There are intriguing questions as to when and how newly learned words are connected to the conceptual-semantic knowledge they refer to. Quite a few proposals have been offered for this aspect of second-language acquisition (e.g., Kroll and Stewart, 1994; Dijkstra and van Heuven, 2002). One problem that hampers the study of foreign-language vocabulary acquisition is that it mainly takes place in situations that do not provide adequate control over the input, the learning context, and many other potentially confounding variables that influence learning success.

For these reasons, researchers are increasingly turning to studying foreign-language learning with what are called novel-word learning paradigms. Common to these paradigms is that the learning input and method, stimulus materials, and external influences can all be kept under much stricter experimental control than in natural learning or in classroom situations. For example, entirely novel words are used instead of existing foreign words, to make sure that there is no overlap between the novel and native language word-forms. While learning with this approach may be ecologically less valid, many confounding influences can be excluded, allowing for clearer conclusions. The experimental manipulation of the learning process further makes it easier to relate the observed effects to the actual learning experience.

In recent novel-word learning studies, words and meanings were associated with rather different methods, such as presenting novel words together with definitions (e.g., Clay et al., 2007; Tamminen and Gaskell, 2013), associating novel words and their concepts by means of pictures (e.g., Yu and Smith, 2007; Dobel et al., 2010), and presenting novel words at the end of meaning-constraining sentences (Mestres-Missé et al., 2007; Borovsky et al., 2010, 2012). Common to these methods is that the word-concept links are established within a rich semantic context and with a salient focus on word meanings. Likewise, tests of these novel links also take place in contexts in which semantic processing is a major element of the task.

To test whether effective word-to-concept links have been established, different speeded and non-speeded tasks have been employed, such as object naming (Breitenstein et al., 2005), translation matching (Dobel et al., 2010), or semantic priming (Dobel et al., 2010; Tamminen and Gaskell, 2013). Results from these studies have shown that such links are indeed established and that these links are also evident when interacting with stimuli that were not presented during learning. However, both the more explicit, non-speeded tasks as well as the semantic priming paradigm are known to be susceptible to strategic manipulations (e.g., Neely, 1991), thus rendering it unclear as to how automatic the activation of the novel word's meaning actually is. Results from the Stroop task (Stroop, 1935; MacLeod, 1991), in contrast, are known to be much more robust against such manipulations. This makes the task a good one to test for at least some components of automaticity in the access process for word meanings^1^ (Moors and De Houwer, 2006). The Stroop task thus promises to be an excellent extension to previous studies, because it allows to assess whether reading a novel word will automatically activate its meaning. Surprisingly, there seems to be only one word-learning study (Altarriba and Mathis, 1997) that made use of the Stroop task to test newly learned links between unfamiliar words and color concepts, and even the results from this study offer only limited conclusions with regard to the automaticity of semantic activation in novel words (see below).

The main focus of the present study is thus (1) to link novel words with familiar concepts within a semantically poor learning context, without an explicit focus on semantic processing and (2) to assess whether this learning nevertheless results in stable links between novel words and their meaning, to the extent that this meaning is automatically activated when merely reading the novel word. Because consolidation effects have been observed in several recent word-learning studies (e.g., Dumay and Gaskell, 2007, 2012; Davis et al., 2008), a further aim of this study is to test whether the establishment and availability of such semantic links in any way depends on an opportunity for memory consolidation.

During learning, novel words were directly paired with L1 (German) color words in a statistical association procedure adapted from Breitenstein and Knecht (2002). In our version of the paradigm, pairs of novel words and native color words are presented—some pairs representing correct matches, some not—such that correct word-word links can only be derived over time, on the basis of co-occurrence frequencies. Importantly, participants are merely instructed to decide whether the novel and native word of the current pair match or not (by pressing one of two buttons)—no semantic processing of the word stimuli is required (but not explicitly prevented). This simple instruction and the fact that no explicit feedback is given make it a procedure of low cognitive demand (e.g., see Clay et al., 2007, for a more explicit procedure, and Kachergis et al., 2013, for an interactive approach). Given that the words are not paired directly with a perceptual representation of their to-be-learned color concepts (e.g., a color patch, a color-related object), any connection between the novel word and the color concept can only be drawn indirectly, via the native color word. The amount of exposure can be easily quantified and manipulated, because novel and native words are associated in a systematic fashion. Likewise, learning progress can be continuously monitored, because a matching judgment is required in every trial. This paradigm has been successfully employed in a number of studies to associate novel words with pictures of existing concepts, using both spoken novel words (Breitenstein et al., 2005, 2007; Yu and Smith, 2007; Dobel et al., 2010; Liuzzi et al., 2010; Freundlieb et al., 2012) and written novel words (Laeger et al., 2014). To our knowledge, our implementation is the first to associate word-word pairs instead of word-picture pairs based on this statistical procedure.

In the typical modern version of the Stroop task, participants name (or indicate by button press) the ink color of a presented word. This response is slowed down if the word's meaning is incompatible with the ink color (e.g., ink color is *red*, but word is BLUE). Thus, the word's meaning interferes with task performance, although the task does not require any processing of the word's meaning. Apparently, reading the word activates the conceptual representation associated with that word. This ability of the Stroop task to reveal automatic semantic activation in such an indirect way promises to be an excellent test for whether, how fast, and how strongly, novel words are linked to their assigned color concepts.

Many studies have investigated how color words from a second language (L2) compare to L1 color words in the Stroop task, with the typical result that a substantial, but smaller interference effect is found in L2 compared to L1 color words (e.g., Preston and Lambert, 1969; Chen and Ho, 1986; Sumiya and Healy, 2004; earlier work reviewed in MacLeod, 1991). Even if the L2 is well-established and participants report equal levels of competence in both languages, the effect is larger in color words from the language that is dominant in everyday use (Altarriba and Mathis, 1997). Stroop effects of comparable size in the speaker's two languages are only found when both usage and competence are equally high (e.g., Mägiste, 1984).

As mentioned earlier, we are aware of only one published experiment in which a group of participants learned the set of novel color words immediately before the Stroop test (Experiment 2 in Altarriba and Mathis, 1997). In this experiment, monolingual English-speaking participants were trained with a set of four Spanish color words and subsequently further familiarized with these words in a series of quizzes. The quizzes involved rehearsing the new lexical link (e.g., matching Spanish to English color words) as well as the new semantic link (e.g., matching the Spanish words to color patches or to compatible objects: *amarillo* [yellow] goes with the school bus). These Spanish words, along with the English translations, were then entered into a Stroop task, in which the ink color had to be named using English color terms. In the English trials, naming latencies between congruently and incongruently colored words differed by 112 ms. Importantly, there was a similar but smaller difference in the Spanish trials (52 ms), indicating that the incompatibility between the word meaning and the verbal response slowed down color naming even with these newly learned words as distractors (Altarriba and Mathis, 1997).

These results are remarkable as they show that, even after a short learning session, the newly learned L2 words have already been sufficiently learned as to interfere with a task that does not explicitly require processing of word meaning. However, some features of this study hinder a full assessment of the power of the underlying semantic learning mechanisms. First of all, the color words of both languages have some phonological and orthographic overlap (e.g., *r*ed—*r*ojo, ye*llow*—amari*llo*) that may have artificially increased the L2 effect (cf. Sumiya and Healy, 2004). Second, given that the experiment was performed in the United States, it is also likely that participants, even though monolingual speakers of English, had some familiarity with the Spanish color words. Finally, the experiment required English color words as responses, which were the same color words that were repeatedly presented with the Spanish words during learning. One could argue that the observed interference was not between the novel words and their meanings, but between the novel words and the required English responses, as these links had been intensely rehearsed during learning (cf. the analogs discussion in the semantic priming literature on the differentiation between genuine semantic priming and priming by association, e.g., Lucas, 2000; Tamminen and Gaskell, 2013).

Hence, in our experiment, the stimuli and parameters of the Stroop task are chosen in such a way that these alternative explanations can be excluded. First, pseudowords instead of existing words are used to serve as to-be-learned color words. This is done to avoid any phonological/orthographic overlap between the L1 and the new color words, and to exclude that participants are familiar with any of the new words. Furthermore, the response format during the Stroop task is changed from verbal responses (color naming) to manual responses (color-matching): Participants indicate the ink color of the presented color-word stimulus by pressing one of four colored buttons. As the buttons are only present during the Stroop task, participants cannot learn any word-response associations beforehand. Consequently, a congruency effect in the Stroop task cannot be explained by a word-response association stemming from the learning phase.

The manual response format offers a further advantage over color naming. Although covert naming cannot be excluded (see e.g., Lupyan, 2012), lexical access is not even necessary to perform the Stroop task. Participants can simply rely on matching the presented ink color to the color of the corresponding button for correct responses. Consequently, this task should make it easier to ignore the presented word and its meaning. Indeed, in the native-language Stroop literature, the manual Stroop effect is usually substantially reduced relative to the verbal Stroop effect (about half the size, MacLeod, 2005). Moreover, Sharma and McKenna (1998) showed that, in contrast to verbal responses, there is no interference component in the manual response format that can be attributed to the word status of control items (that is, they found that a manual color-matching response to an XXXX letter string is as fast as a manual response to a color-unrelated existing word such as CHIEF). This in turn suggests that the manual response format more clearly captures the semantic component of the Stroop effect. In sum, the manual response format offers a stronger test for semantic learning of the novel color words.

Taken together, the adaptations we introduced to the original learning and testing paradigm provide a strong test of the power of semantic novel-word learning and of the automaticity of the resulting memory traces.

A further aim of our study was to test whether the establishment and availability of such semantic links depends on an opportunity for memory consolidation. In most studies that used our variant of a statistical learning procedure, learning took place over a number of consecutive days, and the crucial test of semantic integration was performed after the learning phase had been completed (e.g., Breitenstein et al., 2007; Dobel et al., 2010; Liuzzi et al., 2010; Freundlieb et al., 2012). With such designs, there is ample opportunity for consolidation, and it is not known whether effects obtained after 4 or 5 days of learning would also be present immediately after learning. However, in several other word-learning studies that used more targeted paradigms, clear effects of memory consolidation on word learning were found (e.g., Gaskell and Dumay, 2003; Bowers et al., 2005; Clay et al., 2007; Dumay and Gaskell, 2007; Tamminen et al., 2010; Tamminen and Gaskell, 2013; Bakker et al., 2014; but see Coutanche and Thompson-Schill, 2014; Kapnoula et al., 2015). It was further shown that, while consolidation may also happen during time awake (Walker, 2005; Lindsay and Gaskell, 2013), consolidation of novel words is strongest during sleep (Dumay and Gaskell, 2007; Henderson et al., 2012). There is also evidence that these consolidation effects are directly related to electrophysiological patterns of brain activity during sleep, such as sleep spindles and slow-wave activity (Tamminen et al., 2010, 2013).

Davis and Gaskell (2009) offered an explanation of the word-learning data based on the more general theory of Complementary Learning Systems (CLS; McClelland et al., 1995). According to their account, word learning is based on two separate neural systems, namely a fast-learning but temporary memory system involving the medial temporal lobe (particularly the hippocampus), and a slower-learning but longer-lasting neocortical memory system. Novel lexical entries are thought to rely initially on hippocampal mediation, with this reliance diminishing only some time after initial encoding, by means of interleaving novel and existing memories (possibly via hippocampal memory replay: Rasch and Born, 2008). Thus, novel lexical entries are thought to fully interact with existing neocortical memories only after they have been consolidated, avoiding the danger of catastrophic interference (McCloskey and Cohen, 1989).

Many of the studies that focus on consolidation included learning of novel word-forms and tested whether and when the novel words showed lexical competition effects with existing neighbors. Only a few looked at how acquiring the meaning of a novel word might be influenced by consolidation (e.g., Clay et al., 2007; Tamminen and Gaskell, 2013). The latter studies also showed evidence for consolidation, but the results were less clear than in the lexical-competition studies. Thus, further research is certainly warranted to identify necessary conditions for consolidation effects in semantic word learning.

Here, as the exact mechanism of any consolidation effects was not a focus of our study, a simple method for testing consolidation was selected: the set of novel words was split in half, and the two resulting sets of words were tested at different delays after learning. With this design, we are able to capture basic effects of consolidation, but not specific effects of sleep.

In the following, results from three experiments are reported. In all experiments, participants could associate novel words with L1 color words, by means of the above-described word-word pairing procedure. These words were then entered into a Stroop task, during which participants were instructed to press the button that corresponded in color to the ink color of the presented word. Novel words were presented either in their congruent (“learned”) or in an incongruent ink color. To capture the potential influence of memory consolidation, different subsets of the learned words were tested either immediately after learning and/or a day later.

Experiment 1 assessed whether newly learned color words would show any Stroop effects at all, immediately or a day after learning. To obtain a direct quantitative comparison of the effect sizes in the native and in the novel words, the novel words were intermixed with (L1) German color words. In Experiment 2, novel words were again tested alongside their German counterparts, but after a much shorter learning phase, and control trials were added to assess facilitation and inhibition components of the Stroop effect. Experiment 3 returned to the design of Experiment 1 and tested whether removing the German color word trials from the Stroop blocks affected the basic novel-word Stroop effect. To assess consolidation effects in more detail, this third experiment also included a second group of participants who received their first Stroop block only on the second day, 24 h after learning.

## Experiment 1

Experiment 1 was designed to test whether novel color words are sufficiently integrated into lexico-semantic memory to produce Stroop congruency effects within 24 h of learning. In a brief learning session, novel words were associated with native color words and subsequently tested in a manual Stroop task. To assess potential effects of memory consolidation, half of the novel color words were tested immediately after learning, the other half 24 h later.

### Materials and methods

#### Outline

**Figure 1:**
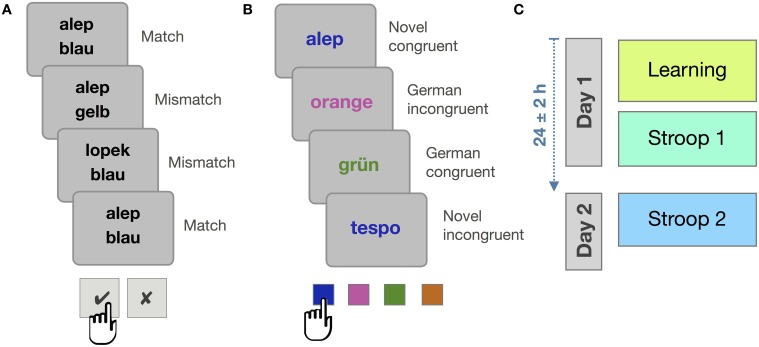
**Overview of Experiment 1**. **(A)** Statistical learning principle: While match and mismatch trials appear equally often, some novel words are paired frequently with a particular native language color word (illustrated here for the pair of *alep* and *blau* [blue]). **(B)** Stroop task: Example stimuli for the four conditions. **(C)** The order of tasks.

Experiment 1 was divided into two sessions, spaced approximately 24 h apart (see Figure 1C for an overview). Session 1 consisted of two parts: (a) Statistical learning of 10 novel words each paired with a German color term, with both novel and German words printed black (*learning phase*); (b) manual Stroop task with a subset of four novel color words and their German translations as stimuli (*Stroop 1*). Session 2: manual Stroop task, with a different subset of four novel color words and their German translations as stimuli (*Stroop 2*). In the manual Stroop tasks, participants had to press one of four colored buttons that matched the ink color of the novel or German word on the screen. To minimize effects of verbal short-term memory, a crossword puzzle separated the learning and test phases on Day 1.

#### Participants

Twenty-four native speakers of German, most of them students, took part in the experiment (21 female; age range: 19 to 28 years, *M* = 21.25, *SD* = 2.33). Participants reported to have no color vision deficiency and had normal or corrected-to-normal visual acuity. They gave their written consent and received course credit or 9 €. All experiments reported here complied with the ethical standards formulated by the Ethics Committee of the Psychology department, University of Münster.

#### Materials

Four focal colors (*red, green, blue, yellow*) and four subordinate colors (*violet, orange, pink, brown*) were selected, as well as *black* and *white*. Except for the latter two, which were included merely to increase the size of the learning set, all colors were used as “ink” colors in the Stroop task. Two different subsets of four colors were used for the Stroop tasks on Day 1 and on Day 2. The two subsets were composed so as to keep the four colors within a set sufficiently discriminable (Set 1: *red*-*yellow-violet-brown*, Set 2: *green-blue-pink-orange*). The two subsets were identical for all participants, but the assignment of the subsets to the two Stroop sessions was counterbalanced between participants.

The 10 corresponding German color words were: *rot* (red), *gelb* (yellow), *blau* (blue), *grün* (green), *lila* (violet), *orange* (orange), *pink* (pink), *braun* (brown), *schwarz* (black), and *weiß* (white). These were used for novel word to color word associations during the learning phase, and except of the latter two, as word stimuli during the Stroop blocks.

Twenty-five nonwords (e.g., *alep, fupo, lopek)* from an existing corpus (Breitenstein and Knecht, 2002) served as novel words in the learning and the Stroop tasks. They are 4–5 letters long and do not elicit any particular lexical associations, as rated by an independent sample (see Breitenstein and Knecht, 2002, for details on word generation and selection criteria). The nonwords are easily pronounceable for native German speakers. Because of their common bi-syllabic structure and simple vowel-consonant alternations, they can be classified as stemming from a common vocabulary of an unknown language. Ten of these nonwords were selected to serve as to-be-learned color names, from which three different sets of novel word to color word assignments were constructed (see *Supplementary Materials*). We made sure that there was no phonological or graphemic onset or offset overlap between selected nonwords and their corresponding German color names within each list. The remaining 15 of the 25 nonwords served as fillers during statistical learning. For practical purposes, we will henceforth use the generic term *Language* to differentiate the sets of German and novel words.

#### Experimental procedure

The experiment was conducted using DMDX software (Forster and Forster, 2003) running on a Windows PC. Stimuli were presented at an eye-to-screen distance of about 60 cm on a 17″ LCD monitor running at 120 Hz. Stimuli appeared on a gray background (RGB values: 210-210-210). Words appeared in lower case *Arial Bold* font, subtending a maximal visual angle of about 3.5° horizontally and 1° vertically. Responses were recorded using a standard Windows keyboard connected via a USB port.

#### Learning procedure

The learning paradigm was adapted from the statistical learning procedure described by Breitenstein and Knecht (2002). During the learning phase, pairs of words were presented on a computer screen. Each pair consisted of a novel word and a German color word. On each trial, a fixation cross appeared centrally for 200 ms, followed by one of the novel words in black font, just above the center. 250 ms later, a German color word was added to the display, just below the center, and also in black. The two-word display remained on the screen for 1500 ms. From the onset of the second word, participants had a 1800 ms time window to decide whether the two words belonged together or not, pressing the right *shift*-key to indicate that the words belong together, or the left *shift*-key to indicate that they do not. Within the learning block, matching and mismatching word pairs appeared equally often (cf. Figure 1A).

Participants were informed beforehand that it was initially impossible to tell whether a pair matched or not, but that during the course of the learning phase, the more frequent co-occurrence of some word-word pairs would help discriminate matching from mismatching pairs. No trial feedback was given except if the participant failed to come up with a response in time, in which case the words “*Zu langsam!*” (= too slow) were presented at the bottom of the screen for 600 ms. After the button press or the time-out feedback, the next trial started after a random delay between 100 and 400 ms.

The statistical learning principle was implemented in the following manner (see also Table 1): During the learning phase, each German color word was presented 24 times with its to-be-associated novel word (*match* trials), and once with each of the remaining 24 novel words (*mismatch* trials). Of the 24 novel words from the mismatch trials, nine were from the other novel words of the learning set (i.e., novel words to take on the meaning of a different color). The remaining mismatch words were novel words that appeared in mismatch trials only and were not systematically associated with any particular meaning. Thus, over the course of the learning phase, participants could find out the matching word-word pairs only by exploiting the frequency of couplings.

**Table 1:** **Frequencies of word pairings during statistical learning of Experiment 1**.

	***gike***	***dufa***	***alep***	***tespo***	***ekir***	***siba***	***eftu***	***dapi***	***fupo***	***rukri***	**+ 15 Filler nonwords**
rot	24	1	1	1	1	1	1	1	1	1	15 × 1
gelb	1	24	1	1	1	1	1	1	1	1	15 × 1
blau	1	1	24	1	1	1	1	1	1	1	15 × 1
grün	1	1	1	24	1	1	1	1	1	1	15 × 1
lila	1	1	1	1	24	1	1	1	1	1	15 × 1
orange	1	1	1	1	1	24	1	1	1	1	15 × 1
pink	1	1	1	1	1	1	24	1	1	1	15 × 1
braun	1	1	1	1	1	1	1	24	1	1	15 × 1
schwarz	1	1	1	1	1	1	1	1	24	1	15 × 1
weiß	1	1	1	1	1	1	1	1	1	24	15 × 1

*Numbers indicate how often each German color word (left column) and a novel word (top row) were presented together during learning. The right column represents the 15 additional pseudowords that were included to obtain an equal number of match and mismatch trials. Each of these additional 15 pseudowords appeared once with every German color word. The assignments of novel words to color words were swapped between participants (there were three different versions)*.

The learning phase consisted of 480 trials and lasted about 22 min. It was subdivided into 4 blocks of 120 trials each, separated by three 30-s breaks. Trials were presented in different random order for each participant, with the constraint that each 120-trial block contained 6 match and 6 non-match trials for each of the German color words. After the learning phase on Day 1, participants filled out the crossword puzzle (duration approx. 5 min.), after which the Stroop task of Day 1 followed.

#### Stroop task

Immediately after the crossword puzzle and again at the beginning of the second day's experimental session, participants took part in a Stroop block. The Stroop tasks of Day 1 and Day 2 were identical except that different sets of four colors were used on each day, along with the corresponding German and the learned novel color words.

In the Stroop task, words were presented one at a time: either a German color word or a novel color word. These words were printed in one of the four ink colors assigned to that session, yielding congruent and incongruent combinations of ink color and word meaning (see Figure 1B). Each trial began with the presentation of a fixation cross that stayed on the screen for 200 ms and was followed by a word presented centrally for 150 ms. Participants were to indicate the ink color of the word by pressing the correspondingly colored response button as quickly as possible. Four buttons of the PC keyboard were used (“y” “x” “,” and “.” on the German layout), marked by correspondingly colored stickers. Participants were to use their left and right middle and index fingers to indicate the ink color the word had been presented in, ignoring the word's meaning. Color-to-button assignments were switched between participants. Participants were given 1800 ms to respond. Feedback was given on the screen for all responses (*Richtig!* = correct, *Falsch!* = incorrect, *Zu langsam!* = too slow). A blank screen (random duration between 850 and 1150 ms) concluded each trial.

For the Stroop task, we selected only one incongruent ink color for each German or novel color word: e.g., we presented *gike* either in red (congruent) or in yellow (incongruent), not in the ink colors violet and brown that also appeared during the same block (see Table 2). The reason for this deviation from the classic Stroop design is the following: In a typical native-language four-colors Stroop task (e.g., with colors red, green, blue, yellow), each color word is presented three times as often in the congruent version (*red* printed in red) as in each of the three possible incongruent versions (*red* printed in green, blue, or yellow), such that congruent and incongruent trials occur equally often. However, if we had presented the novel-word Stroop trials according to this scheme, participants would have had an additional opportunity to learn the correct novel-word-to-color couplings (because, e.g., *gike*, meaning red, is more often presented in red than in any of the other colors). Moreover, such a presentation scheme would also have provided the opportunity for direct word-response association (e.g., *gike* = second button from left), which would be a severe confound in a manual Stroop task. Schmidt et al. (2007) present evidence for such associative learning within the Stroop task. By presenting the color words in just one incongruent version, we eliminated any opportunity to learn the correct word-color or word-response pairs within the Stroop task. Crucially, this excludes the possibility that subsequent performance differences between congruent and incongruent Stroop trials might be due to or influenced by learning effects during the Stroop task itself. The German color words were presented in the same incongruent color as the corresponding novel color words.

**Table 2:**
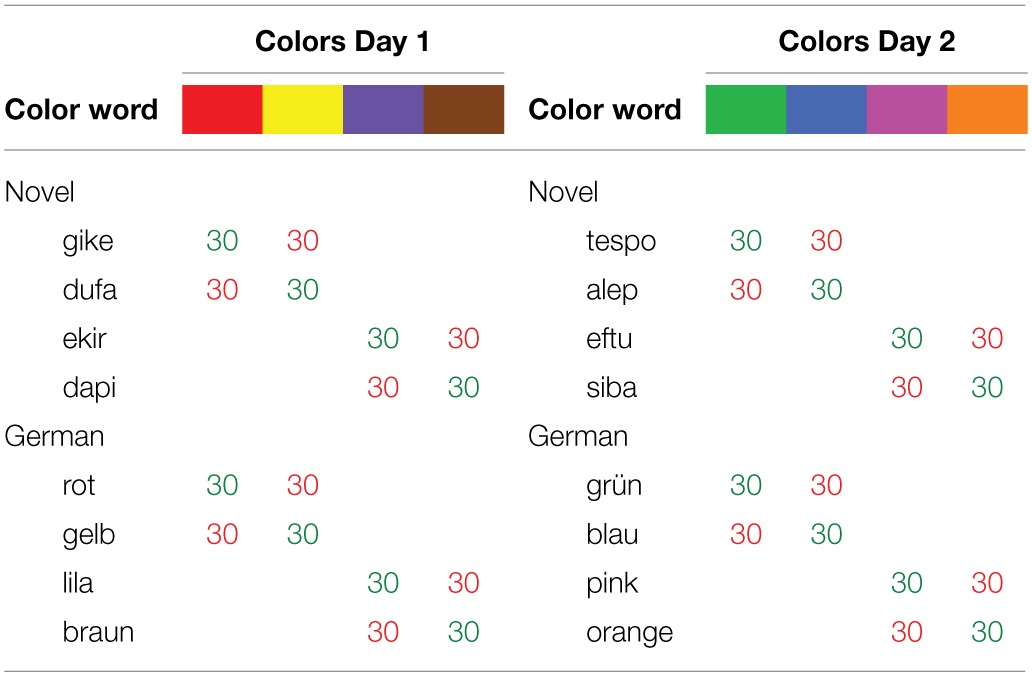
**Overview of color-word stimuli in the Stroop task**.

*The Stroop stimuli can be reconstructed as combinations of ink color (top) and novel or German color word (left). The numbers represent the repetitions of the color-word stimulus during one Stroop block. Green numbers represent congruent stimuli, red numbers represent incongruent stimuli. Half of the participants received the color subsets in reverse order, i.e. the green-blue-pink-orange subset on Day 1 and the other set on Day 2*.

Each of the session's four German and four novel color words was shown 30 times in its congruent and 30 times in its incongruent ink color, yielding 480 trials, which were presented randomly in 4 blocks of 120 trials, separated by breaks of 30 s. The Stroop task lasted about 26 min.

On the second day, 24 ± 2 h after the first session, participants returned to the laboratory to repeat the Stroop task. This second Stroop task included the remaining set of four colors and their corresponding German and novel color words. All other details were identical to the Stroop task on Day 1.

### Results

#### Learning phase

To assess learning success, the percentage of correct responses was calculated for the final block from the learning phase (last 60 trials). Participants reached an average level of 95.1 % [*SD* = 4.4] correct decisions (chance level = 50%; see *Supplementary Material* for learning curves to all three experiments).

#### Stroop task

**Figure 2:**
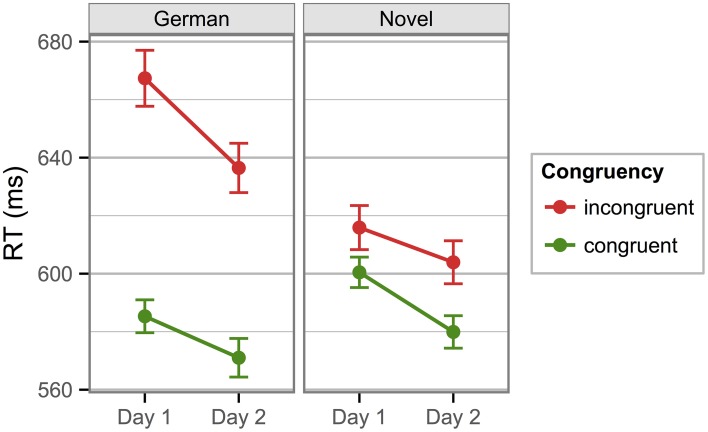
**Mean response times in the Stroop task of Experiment 1**. Error bars here and in the following graphs indicate within-participant standard errors of the mean (Loftus and Masson, 1994; Cousineau, 2005; Morey, 2008).

For reaction time (RT) analysis of the Stroop data, the first 40 trials of each day's Stroop block, error trials, as well as the slowest and fastest 5% of each condition's remaining responses were discarded before calculating mean RTs. On both days and in both stimulus languages, responses to incongruent trials were slower than those to congruent trials, but the effect was larger in the German trials (Figure 2).

A repeated-measures analysis of variance (ANOVA) with factors *Language* (German/Novel), *Congruency* (Congruent/Incongruent) and *Day* (Day 1/Day 2) was calculated to confirm these observations. There were main effects of *Language* (responses to German words were slower than those to novel words), *F*_(1, 23)_ = 28.49, *p* < 0.001, η^2^_p_ = 0.55, and of *Congruency* (responses to congruent stimuli were faster than those to incongruent stimuli), *F*_(1, 23)_ = 95.80, *p* < 0.001, η^2^_p_ = 0.81. The main effect of *Day* just failed significance, *F*_(1, 23)_ = 3.87, *p* = 0.061, η^2^_p_ = 0.14. As indicated by a significant *Congruency* by *Language* interaction, *F*_(1, 23)_ = 65.12, *p* < 0.001, η^2^_p_ = 0.74, the congruency effect was larger for German color words (mean congruency effect over both days: 73 ms) than for novel color words (mean effect 20 ms). The remaining two-way interactions did not reach significance (*Fs* ≤ 1.31, *p*s ≥ 0.264).

To add statistical backing to the visual impression that congruency effects were present at both time points in both stimulus languages, we calculated separate repeated-measures ANOVAs for the German and novel word mean RTs, each including *Congruency* and *Day* as factors. The resulting pattern of effects was identical for both languages. The only significant effect in both cases was the main effect of *Congruency*: German words, *F*_(1, 23)_ = 129.40, *p* < 0.001, η^2^_p_ = 0.85; novel words, *F*_(1, 23)_ = 14.97, *p* < 0.001, η^2^_p_ = 0.39. The main effect of *Day* was marginally significant in both languages: German words, *F*_(1, 23)_ = 4.01, *p* = 0.057, η^2^_p_ = 0.15; novel words, *F*_(1, 23)_ = 3.17, *p* = 0.09, η^2^_p_ = 0.12. The interaction effect was not significant in either of the languages: German words, *F*_(1, 23)_ = 2.01, *p* = 0.170; novel words, *F*_(1, 23)_ = 0.56, *p* = 0.46. Thus, in both stimulus languages, the congruency effect was present on both days and did not change significantly between days.

Despite the fact that in both stimulus languages *Congruency* did not reliably interact with *Day*, there was a Three-Way interaction of *Language* by *Congruency* by *Day* in the overall ANOVA, *F*_(1, 23)_ = 5.69, *p* = 0.026, η^2^_p_ = 0.20. This is explained by the fact that the change of the congruency effect from Day 1 to Day 2 goes in opposite directions in the two languages: There is a decrease of the congruency effect in the German words from Day 1 to Day 2 (from 82 to 65 ms), and an increase of the effect in the novel words (from 15 to 24 ms). Although these changes themselves are not significant (see interaction effects in within-language ANOVAs), the three-way interaction is.

Errors showed a similar pattern as the RTs. A repeated-measures ANOVA with factors *Language, Congruency*, and *Day* on the arcsine-transformed percent error rates revealed significant main effects of *Language, F*_(1, 23)_ = 15.48, *p* < 0.001, η^2^_p_ = 0.40, and *Congruency, F*_(1,23)_ = 12.55, *p* = 0.002, η^2^_p_ = 0.35. Neither the main effect of *Day* nor any of the interactions reached significance (all *F*s < 2.29, all *p*s > 0.143). Averaged over the two sessions, the mean percent error rates were (*SD*s in brackets): German congruent, 5.71 [3.80], incongruent, 7.84 [5.04], novel congruent, 4.91 [3.76], incongruent, 6.48 [4.03].

### Discussion

Experiment 1 was designed to test whether novel words that have recently been associated with native color words via lexical association are already able to produce a congruency effect in the Stroop paradigm. The response-time findings show that this is indeed the case: Immediately after learning as well as 24 h later, novel color words generated sizable congruency effects. Given that learning in this experiment consisted of a word-word-association procedure that neither required nor encouraged deep semantic processing of the novel words, the presence of a Stroop effect seems notable. The fact that we see the effect immediately after learning suggests that, under these conditions, consolidation is not necessary for the effect to emerge.

We further found that the change of the congruency effect between the two sessions was not identical in the two stimulus languages: The congruency effect in the German words decreased by 17 ms on the second day compared to the first day's Stroop session, while in the novel words the effect increased by 9 ms. Thus, in both languages, congruency effects are present on both days, but the significantly contrasting pattern of overnight changes in the Stroop effects, signaled by the three-way interaction, points to the possibility that, during the 24 h interval, the two classes of words are processed in a qualitatively different way. Experiment 3 will address the question of time and consolidation effects more directly.

The learning run in this first experiment, although based on a relatively shallow learning task, contained a large number of trials per word and thus resulted in a classification performance that approached ceiling levels. It is therefore unclear whether the novel word congruency effect crucially depends on such a large number of learning trials or whether a significant reduction of the trial number will lead to a qualitatively similar result.

Furthermore, so far the Stroop sessions only contained congruent and incongruent trials but no neutral control stimuli, rendering it impossible to clearly identify the effect as facilitatory, inhibitory, or a mix of both. In native-language Stroop, these two main components (facilitation and inhibition) can indeed be distinguished (e.g., Redding and Gerjets, 1977). They are respectively defined as the difference in response times between neutral control stimuli and congruent stimuli (facilitation) or between neutral control stimuli and incongruent stimuli (inhibition). While the relative proportions of the components may vary depending on the properties of the neutral stimuli (e.g., Sharma and McKenna, 1998), the interference component is typically substantially larger than the facilitation component (MacLeod, 1991). If the novel word effect were closely linked to the native words effect, then it should at least be similarly divisible into an inhibitory and a facilitatory component.

In Experiment 2, we addressed both the question of learning intensity and the question of whether the novel word congruency effect is composed of facilitation, inhibition, or both.

## Experiment 2

The design of Experiment 2 closely followed that of Experiment 1, but it contained two changes. First, we lowered the number of learning trials per novel word to one third of that from the previous experiment, to test whether the congruency effect in the novel words is obtained even if the classification performance at the end of learning is significantly reduced. Second, to isolate facilitation and inhibition effects, we introduced neutral control stimuli into the experiment, namely names of non-color-related objects.

Because these control stimuli were supposed to serve as a baseline for the respective stimulus language's color words, we introduced control items for both languages: for German color words, a set of not color-related German object names (e.g., *Mappe* [folder]); for novel color words, a further set of novel words that were to become translations of the German object names. The latter were learned in the same manner as the novel color words. Thus, German and novel color words had their own corresponding lexical baselines (the respective object names). The experiment also included a set of non-lexical control stimuli (strings consisting of upper- and lower case X-letters), but because responses to these stimuli did not differ from those to the other (lexical) control items, we will only briefly report the results from this condition.

### Materials and methods

#### Participants

Participants were 41 native speakers of German, most of them students (29 female; age range: 19–42 years, *M* = 24.06, *SD* = 4.68). They reported to have no color vision deficiency and had normal or corrected-to-normal visual acuity. Participants gave their written consent and received course credit or 9 €. One participant's data were incomplete and thus discarded from further analysis.

#### Materials

For this experiment, the same four focal and four subordinate colors as in Experiment 1 were used, omitting black and white from the learning set. For the lexical control condition, we selected eight names of objects that can appear in different colors but are not associated with one color in particular (as rated in an independent sample, e.g., Mappe [folder], Eimer [bucket], see *Supplementary Material*). We also included a non-lexical control condition that consisted of eight different strings made up of upper and lower case X letters (length 3–7 letters, e.g., XxxXX, XxX).

#### Procedure

Laboratory and apparatus were the same as in Experiment 1, as was the overall procedure (cf. Figure 1C).

#### Learning procedure

The eight object names were added to the eight colors, to form a set of 16 concepts for which pseudowords had to be learned. Sixteen pseudowords from the vocabulary described in Experiment 1 served as associates for the colors and object names.

The number of learning trials per color word or object name was reduced to only a third of the previous version. That is, eight match and eight mismatch trials were now presented per concept during learning (instead of the former 24 each). The learning principle remained identical: For the match trials, each native color word or object name was presented eight times with its assigned novel word. For the mismatch trials, each concept was presented once with each of eight other novel words. These were now all taken from the set of the remaining 15 pseudowords that were to attain a meaning (i.e., no additional filler pseudowords were used). To avoid stimulus-specific effects, four different assignments of novel words to native color words or object names were created.

In the learning phase, a total of 256 trials were presented (16 novel words × 16 trials [8 match, 8 mismatch]). These were shown in four blocks of 64 trials, with three 30-s breaks in-between. Trials were presented randomly with the constraint that, per novel word, two match and two mismatch trials were presented per 64 trial block. Trial timing and instructions were identical to those of Experiment 1.

#### Stroop task

The Stroop tasks of Day 1 and 2 closely followed the design from Experiment 1, with the following changes: Apart from German color word and novel color word trials, the Stroop task now also included lexical control trials consisting of the German and the learned novel object names, as well as non-lexical control trials made up of letter X strings. As in Experiment 1, the eight colors were split into two sets, such that four different colors were tested on Day 1 and on Day 2. Likewise, the sets of eight object names and eight strings of the letter X were split into two subsets, tested on either the first or the second day.

The German and novel color words were shown in their natural congruent and in one assigned incongruent version, just as in Experiment 1. Each control stimulus was also shown in only two color versions, to assure that the colors it was presented in were equally predictable as those of the color words. There were altogether 20 different strings in a day's Stroop block (4 German color words, 4 novel color words, 4 German object names, 4 novel object names, 4 letter X strings), each presented in two variants of ink color. The resulting 40 stimuli were each presented 15 times. Thus, over the whole block, 600 trials were presented, in a random order and with breaks after every 120 trials.

### Results

#### Learning phase

To assess learning performance, the percentage of correct responses was calculated for the final block from the learning phase (last 32 trials). Separate values were calculated for the two stimulus types *novel color word* and *novel object name*. Participants reached similar levels of correct decisions for the color words (*M* = 79.0 % [*SD* = 12.15]) and for the object names (77.0 % [13.39]), substantially lower than the final performance in Experiment 1: 95.1 % [*SD* = 4.4].

#### Stroop task

**Figure 3:**
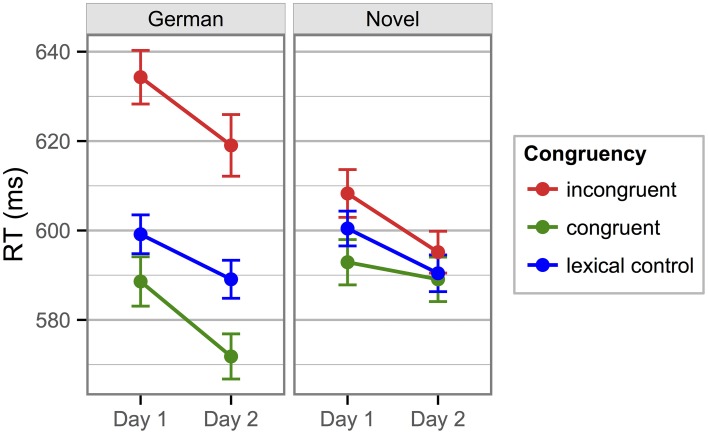
**Mean response times in the Stroop task of Experiment 2**. The respective lexical control stimuli (blue lines) are comprised of German object names (left) and newly learned object names (right). Because the non-lexical control condition (strings of the letter X) had RTs that were practically identical to the lexical control conditions, they are not shown here.

Mean RTs in the Stroop task were calculated as in Experiment 1. For the German words, the expected pattern was observed: a large difference of mean RTs between the congruent and incongruent conditions and a lexical control condition that was situated in between, somewhat closer to the congruent condition than to the incongruent condition. Novel words showed a smaller effect but a similar pattern: congruent trials yielded faster responses than incongruent trials, and lexical control items were situated between the congruent and incongruent conditions (see Figure 3). Mean RTs from the non-lexical control items (the strings of the letter X; not shown in Figure 3) were identical to those from the lexical control items (in line with Sharma and McKenna, 1998) and are therefore not further analyzed (Day 1: *M* = 599 ms [*SD* = 71], Day 2: 589 [65]).

To substantiate these observations, we calculated a repeated-measures ANOVA with factors *Language* (German/Novel)*, Day* (Day 1/Day 2), and *Congruency* (Congruent/Incongruent/Neutral). Greenhouse-Geisser corrected *p*-values are reported where it is warranted by violations of the sphericity assumption. We observed a main effect of *Congruency, F*_(2, 78)_ = 55.46, *p* < 0.001, η^2^_p_ = 0.58, a marginal main effect of *Language, F*_(1, 39)_ = 3.36, *p* = 0.075, η^2^_p_ = 0.08, and a significant *Congruency* by *Language* interaction, *F*_(2, 78)_ = 28.48, *p* < 0.001, η^2^_p_ = 0.42. None of the remaining effects were significant (all *F*s < 2.35, all *p*s > 0.134). As in Experiment 1, we calculated separate follow-up ANOVAs for the two stimulus languages, each incorporating *Day* and *Congruency* as factors, which confirmed that, within both stimulus languages, the only significant effect was the main effect of *Congruency*: German words, *F*_(2, 78)_ = 61.43, *p* < 0.001, η^2^_p_ = 0.61; novel words, *F*_(2, 78)_ = 6.53, *p* < 0.001, η^2^_p_ = 0.14. Additionally, there was a marginal main effect of *Day* in the German words, *F*_(1, 39)_ = 2.92, *p* = 0.095, η^2^_p_ = 0.07. No other effects were significant, *F*s < 1.49, *p*s > 0.229.

Finally, to isolate facilitation and inhibition components of the congruency effect, we calculated separate *F*-contrasts for these effects in each language. Because, in the overall ANOVA, there was no significant interaction involving the factor *Day*, we aggregated the RTs across the two sessions to increase statistical power. For the German words, there were significant effects of facilitation and inhibition: difference neutral—congruent, 13.9 ms, *F*_(1, 78)_ = 10.45, *p* = 0.002, η^2^_p_ = 0.12; difference incongruent—neutral, 32.5 ms, *F*_(1, 78)_ = 112.41, *p* < 0.001, η^2^_p_ = 0.59. For the novel words, there was no significant effect of facilitation, but a significant inhibition effect: difference neutral—congruent, 4.5 ms, *F*_(1, 78)_ = 2.22, *p* = 0.140; difference incongruent—neutral, 6.3 ms, *F*_(1, 78)_ = 10.84, *p* = 0.001, η^2^_p_ = 0.12.

Error rates were similar across all conditions. A repeated-measures ANOVA with factors *Language, Day*, and *Congruency* on arcsine-transformed error rates showed no significant effects (all *F*s < 0.60, all *p*s > 0.443). Averaged across the three congruency conditions and the two days, the percent error rates and standard deviations were similar for the two languages (German: 6.59 [3.18], novel: 6.69 [3.29]).

### Discussion

In Experiment 2, we investigated whether newly learned color words lead to a Stroop effect after a much-shortened learning phase. We also included control stimuli to test whether the novel word Stroop effect is driven by inhibition, facilitation, or a combination of both. Despite a significantly shortened learning phase and the inclusion of control trials, the novel-word Stroop effect was still present, and notably so in the Stroop block immediately after learning. As predicted, the novel word effect was smaller than in Experiment 1 (averaged over the two sessions: 20 ms in Experiment 1 vs. 11 ms in Experiment 2). The fact that the effect size in the German trials was also reduced significantly (from 74 ms in Experiment 1 to 47 ms in Experiment 2) lends some support to the idea that not only the less intense learning but also the inclusion of control trials, and thus the decrease of the proportion of congruent trials (Bugg and Crump, 2012), may have contributed to the reduction in the novel-word effect.

As in our German trials and in the Stroop literature, response times to novel lexical control words were in between responses to congruent and incongruent novel color words. The overall congruency effect was small (11 ms), rendering differentiation between facilitation and inhibition components difficult. Nevertheless, the contrast between the control and incongruent condition shows a significant difference, indicating that the overall effect contains an interference component.

Contrary to Experiment 1, Experiment 2 showed no indication that the time of test (immediately after learning vs. 24 h later) had an impact on how the novel and German stimuli were processed in the Stroop task. Both experiments demonstrated that recently learned novel color words lead to significant Stroop effects, immediately after they have been learned. Note that the novel words were always tested in blocks that also contained the German words they had been associated with during learning. It is easily conceivable that the presence of the German color words in the Stroop blocks helped activating the links between novel words and color concepts. The Complementary Learning Systems (CLS) account of word-learning provides a framework for effects of context on learning and consolidation (Davis and Gaskell, 2009). Before consolidation, novel words are thought to exist only as episodic and context-dependent memory traces. Only after consolidation, that is, after successful transfer of the learned contents from the medio-temporal to the neocortical memory system, do novel lexical traces become independent of the specific learning context.

Evidence on this hypothesis is still sparse, but a relevant study was presented by Tamminen et al. (2012). They taught participants a set of novel, meaning-conveying affixes (e.g., -*nule*) by pairing the affix with an existing word stem (e.g., *buildnule*) and accompanying it with a definition of the composite meaning (e.g., “*buildnule*—someone who is able to build furniture at a remarkable speed”). These affixes showed an immediate advantage in a speeded shadowing task, but only when presented in their trained context (e.g., *buildnule*). This advantage in shadowing performance generalized to untrained word stems and thus to novel contexts (e.g., *sailnule*) only after consolidation. In a non-speeded classification task, however, generalization effects emerged already immediately after training. Thus, their study supports the hypothesis that context-independence of novel lexical items requires memory consolidation, particularly so when the task used to test the novel lexical items requires rapid online processing (Tamminen et al., 2012).

More detailed knowledge about moderating factors is certainly desirable. Deleting the L1 (German-words) context from the Stroop blocks is thus a useful change in design relative to Experiments 1 and 2. Because our experiment is indeed based on a speeded task, we reasoned that the presence of the German words (and thus of additional learning context) in Experiments 1 and 2 may have masked a more pronounced effect of memory consolidation on Stroop performance. We thus wanted to assess whether novel words activate their semantic concepts on their own, without support from the native-language color words. To do so, it is necessary to test novel words in Stroop blocks that do not provide any learning context. This is the key element of Experiment 3.

## Experiment 3

In this experiment, we returned to the design of Experiment 1 with more extensive learning and without control trials. Crucially, novel color words were now tested in the Stroop blocks in isolation, without German color word trials. The stimuli that initially linked novel words and color concepts were thus no longer available during the Stroop test. Apart from the novel words themselves, no further stimuli from the learning context were available. Following the CLS prediction, we hypothesized that, in the absence of further learning context, the Stroop effect in Experiment 3 would only show on the second day, after memory consolidation had taken place.

If we were to indeed find such a pattern, one could still argue that, in the absence of the German words, participants may need more time to familiarize themselves with the task and that a novel word effect might thus appear only after a sufficient number of trials, possibly coinciding with the transition between the two blocks. Therefore, to differentiate between such a practice effect and an effect of memory consolidation, we added a second group of participants that also did two Stroop blocks, but did both of them only on Day 2, 24 h after the learning phase. If this second group showed a Stroop effect only in their second Stroop block, a practice effect must indeed be underlying the hypothesized Group 1 pattern. If however, Group 2 shows the effect already in their first Stroop block, then this difference to the Group 1 pattern must be a consequence of the passage of time, providing an opportunity for memory consolidation.

At the end of the experiment, both groups received a Stroop block with four of the German color words to allow for a numerical comparison of native and novel word effects.

### Materials and methods

#### Participants

Participants were 44 native speakers of German, most of them students (34 female; age range: 19–46, *M* = 24.72, *SD* = 6.55). They reported no color vision deficiency and had normal or corrected-to-normal visual acuity. Participants gave their written consent and received course credit or 9 €. Participants were randomly assigned to the two groups, such that there were 22 participants in each group. The data from three participants had to be discarded because they were either incomplete (2 participants) or because of excessive error rates in the Stroop task (>33% errors, 1 participant). The resulting group sizes used for statistical analysis were 20 (Group 1) and 21 participants (Group 2).

#### Procedure

**Figure 4:**
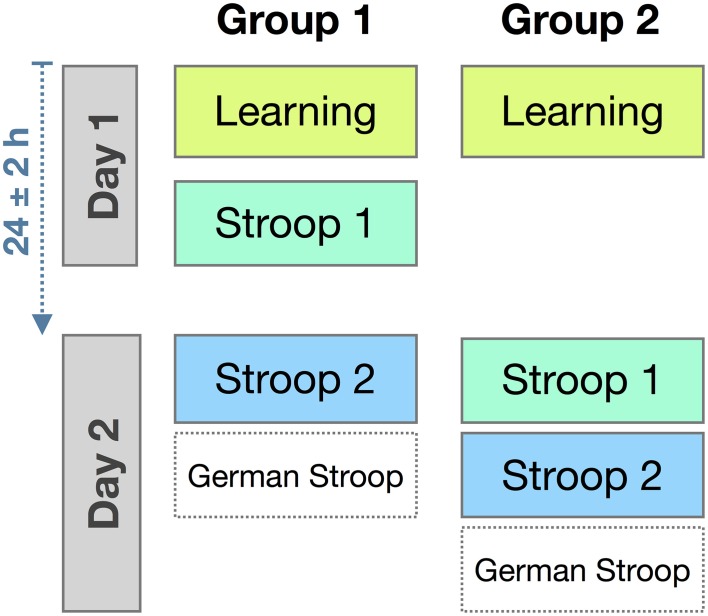
**Time course of tasks for the two groups in Experiment 3**.

Experiment 3 incorporated the same materials and procedures as Experiment 1, except for the following changes: First, from the 480 trials in each of the two Stroop blocks, we removed the 240 trials that contained German color words. The remaining 240 novel color word trials of each Stroop block were presented as in Experiment 1, that is, in random order and with a short break after 120 trials. Second, the time points at which the Stroop blocks were presented were manipulated between groups. Group 1 was subjected to the same temporal procedure as in Experiment 1, that is, with one Stroop block shortly after learning, and one Stroop block about 24 h later. For Group 2 however, only the learning phase was presented on Day 1. The Stroop blocks for Group 2 were both presented only on Day 2 (cf. Figure 4).

Both groups received a block with 240 German color word Stroop trials after the second novel word Stroop block. The four colors for the German Stroop block were those that were used for the second novel word Stroop block, such that the color-to-button assignments of the second block remained valid for the German Stroop block.

### Results

#### Learning phase

Learning performance was assessed as in Experiment 1, separately for the two groups. Participants from Group 1 reached an average of 90.2% [3.06] correct decisions in the final block, those from Group 2 reached 94.5% [2.39]. This difference was not statistically significant, Welch's *t*-test: *t*_(25.79)_ = 1.35, *p* = 0.188.

#### Stroop task

Mean RTs were calculated as before. RTs in the final German Stroop block showed the expected effect: Group 1 had a congruency effect of 77 ms (incongruent 654 ms [60], congruent 577 [20]), Group 2 had an effect of 59 ms (incongruent 640 [53], congruent 581 [21]). Because the German words block was merely included to compare the size of native and novel effects, the data were not analyzed statistically.

**Figure 5:**
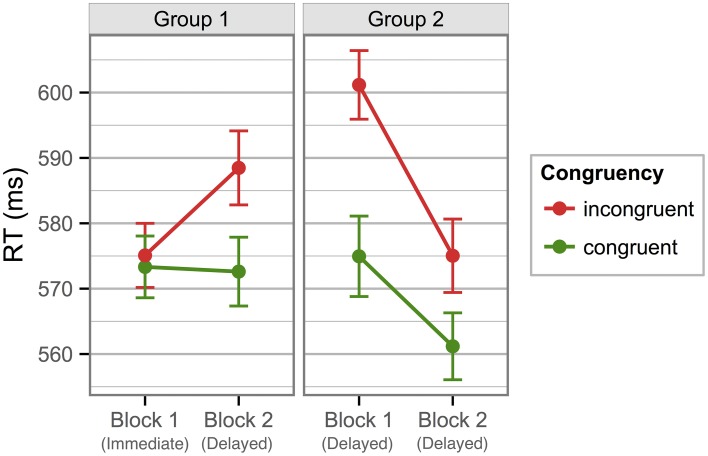
**Mean response times to novel color words in the Stroop blocks of Experiment 3**. In contrast to the previous two experiments, only novel words were included in these Stroop blocks. The left panel shows data from Group 1, which did their first Stroop block immediately after learning and the second block 24 h later. The right panel shows data from Group 2, which did both Stroop blocks on Day 2.

Mean RTs from the novel-word Stroop blocks showed that only Group 2, tested 24 h after learning, had a congruency difference in their Block 1 (of 26 ms). Group 1, who performed their first Stroop block immediately after learning, showed no such effect (congruency difference = 2 ms). Nevertheless, in the second Stroop block, which both groups performed on the second day, both groups showed a clear Stroop effect, which was furthermore identically sized (14 ms; see Figure 5).

These observations were confirmed in a mixed repeated-measures ANOVA on the novel word mean RTs that included the within-participant factors *Congruency* (Congruent/Incongruent) and *Stroop Block* (Block 1/2) and the between-participants factor *Group* (Group 1/Group 2). The only significant main effect was that of *Congruency, F*_(1, 39)_ = 28.79, *p* < 0.001, η^2^_p_ = 0.42. The *Group* by *Congruency* interaction was also significant, *F*_(1, 39)_ = 4.36, *p* = 0.043, η^2^_p_ = 0.10, and so was the *Group* by *Stroop Block* interaction, *F*_(1, 39)_ = 5.54, *p* = 0.024, η^2^_p_ = 0.12. Crucially, there was a three-way interaction of *Group* by *Congruency* by *Stroop Block, F*_(1, 39)_ = 9.13, *p* = 0.004, η^2^_p_ = 0.19, indicating that the development of the *Congruency* effect over the two *Stroop Blocks* differed between *Groups*. All other effects were non-significant (*F*s ≤ 1.49, *p*s ≥ 0.230).

To further explore the three-way interaction, we calculated separate ANOVAs for the two Stroop blocks, both including the between-participants factor *Group* and the within-participants factor *Congruency*. In the first Stroop block, the main effect of *Group* was not significant, *F*_(1, 39)_ = 0.40, *p* = 0.531. But the main effect of *Congruency* and the *Congruency* by *Group* interaction were significant, *F*_(1, 39)_ = 17.54, *p* < 0.001, η^2^_p_ = 0.31, and *F*_(1, 39)_ = 13.41, *p* < 0.001, η^2^_p_ = 0.26, respectively. The latter result confirms that the two groups clearly differed in their response patterns in the first block. With the data from the second Stroop block, only the main effect of *Congruency* reached significance, *F*_(1, 39)_ = 17.12, *p* < 0.001, η^2^_p_ = 0.31 (the other two effects: *F*s ≤ 0.32, *p*s ≥ 0.575), suggesting no difference in the response patterns for the two groups.

Because final learning performances of the two groups differed numerically (although not statistically), we wanted to make sure that this difference did not affect the pattern of the Stroop RTs. We therefore recalculated the Stroop RT analysis in the following manner: We removed the data for the five best-performing learners of Group 1 and the five worst-performing learners of Group 2, such that we obtained closely matching learning curves and final discrimination performances between the two groups. We then recalculated the main ANOVA that included all three experimental factors. The pattern of results did not change, with the effect size of the critical three-way interaction actually increasing: *F*_(1, 29)_ = 10.41, *p* = 0.003, η^2^_p_ = 0.21.

There were again few differences between congruency conditions in the error rates. In the first Stroop block, error rates seemed to mirror the result from the RTs: There was no congruency effect in Group 1's first Stroop block (% errors congruent *M* = 8.50 [*SD* = 5.88], incongruent 7.67 [5.33]), but there was one in Group 2 (congruent 4.84 [3.38], incongruent 7.54 [3.82]). In the second Stroop block, neither group showed congruency differences in the error rates (Group 1: congruent 6.88 [6.39], incongruent 6.42 [4.95], Group 2: congruent 4.25 [3.04], incongruent 4.92 [3.40]). A repeated-measures ANOVA on the arcsine-transformed error rates with factors *Congruency, Block*, and *Group* showed a main effect of *Block, F*_(1, 39)_ = 16.59, *p* < 0.001, η^2^_p_ = 0.30, indicating an overall reduction of errors in the second block. There was also a *Congruency* by *Group* interaction, *F*_(1, 39)_ = 5.47, *p* = 0.002, η^2^_p_ = 0.12, reflecting that Group 2 made more errors in the incongruent condition. This interaction effect seems to be particularly driven by Group 2's large congruency difference in Block 1, but the three-way interaction of *Congruency, Block*, and *Group* just failed significance, *F*_(1, 39)_ = 3.59, *p* = 0.065, η^2^_p_ = 0.08.

### Discussion

In the third experiment, we tested whether the novel-word Stroop effect depended on the presence of German words during the Stroop test. We therefore removed the German words from these blocks and otherwise repeated Experiment 1. We added a between-participants manipulation to differentiate predicted consolidation effects from effects of mere temporal order. One group of participants performed the first Stroop block immediately after learning and the second Stroop block about 24 h later. A second group of participants had no Stroop block immediately after learning, but rather performed both blocks on the second day.

Results show that in Group 1, the Stroop effect was not present in the block that was administered immediately after learning but only on the second day. In Group 2, with both Stroop blocks on the second day, the effect was present already in the first Stroop block. These results lead to two important conclusions: First, the novel word effect can be observed even when no German color words are included in the Stroop blocks. Second, the differing results between the two groups indicate that, in the absence of the native-language words, the effect arises only after a period that allows memory consolidation.

## General discussion

In three experiments, we tested the semantic links of novel color words that had been associated with color concepts through lexical association with native language (German) color words. To assess which conditions are necessary for semantic learning, the learning and test phases were realized such that they minimized semantic processing. In Experiment 1, novel words were associated with native-language color words until almost perfect discrimination performance. They were then entered into the Stroop task together with German color words. We observed substantial novel word Stroop effects both immediately after learning and 1 day later. A significant three-way interaction indicated that the reduction of the effect from Day 1 to Day 2 in the German words contrasted significantly with a simultaneous increase of the effect in the novel words, thus suggesting an influence of memory consolidation. In Experiment 2, learning intensity was considerably reduced and neutral control stimuli were added to the Stroop blocks. We again observed substantial Stroop-congruency effects directly after learning and 24 h later. A detailed analysis including the control condition showed that interference made up a significant portion of the novel-word Stroop effect. In Experiment 3, we repeated Experiment 1, but crucially removed the German words from the Stroop task, so that the novel words were now tested without any L1 context from the learning phase. The novel-word Stroop effect was now not observed immediately after learning, but only 24 h later. Results from a second group that had a different time course of Stroop blocks showed that the delayed emergence of the effect in Group 1 is not due to a simple build-up or training effect from one block to the next. Rather, it must be related to the temporal distance between learning and test—that is, to memory consolidation.

It should be stressed again that semantic processing, though still possible and likely given the explicit setting, was not necessary for correct task performance, neither during learning (lexical association) nor in the timed memory test (color-matching Stroop task). Given the potential of shallow association, it is surprising that a congruency effect emerged at all. This is broadly consistent with a number of recent studies (Breitenstein et al., 2007; Clay et al., 2007; Mestres-Missé et al., 2007; Borovsky et al., 2010, 2012; Dobel et al., 2010; Tamminen and Gaskell, 2013) in showing a rapid and effective link-up of novel words with an assigned concept—in our case, even despite an intentionally shallow learning experience and an impoverished semantic context. While in almost all earlier studies, novel words were associated with concepts either via pictures or in semantically elaborate contexts (e.g., with definitions or in sentence contexts), here, meanings were introduced merely via lexical association with an L1 word (see also Experiment 3 in Duyck and Brysbaert, 2004). The emergence of Stroop effects in this situation shows that, even when potential meanings for novel words can only be indirectly derived via the L1 word, these novel words may nevertheless activate their associated meanings early on.

Note that the novel-word Stroop effect obtained in our study is not so much based on priming but rather on interference (cf. Experiment 2). This fits with results from the native-language Stroop literature. To our knowledge, there are hardly any studies that show a semantic interference effect for recently learned words. Clay et al. (2007) used a picture-word interference paradigm that generally produces interference of semantic relatedness between pictures and distractor words (e.g., picture of a cat that has to be named, distractor word “dog”), and they found a similar interference effect in newly learned words. This and our current result support the conclusion that semantic novel-word effects are not constrained to facilitation and priming paradigms, but generalize also to semantic interference paradigms such as picture-word interference and Stroop. Whereas priming is often considered to have automatic as well as controlled components (Neely, 1991), this Stroop interference effect strongly suggests that reading a novel word co-activates its recently learned meaning in an automatic fashion, even when the semantic context is highly impoverished (only 4 colors in a test block) and when a meaning for novel words is not needed to fulfill the task.

Perhaps the most interesting aspect of our results is that the opportunity for consolidation affected Stroop performance, and that this consolidation effect was further moderated by context, that is, by the presence of German color word trials in the Stroop task. This impact of memory consolidation on the integration of novel words fits with data from word-learning studies on the learning of word forms only (e.g., Gaskell and Dumay, 2003; Dumay and Gaskell, 2007, 2012; Bakker et al., 2014) or on the acquisition of form and meaning (Clay et al., 2007; Tamminen et al., 2012; Coutanche and Thompson-Schill, 2014). The data from Experiment 3 in particular demonstrate that memory consolidation is relevant for associating novel words with meaning, not only for integrating novel *word forms* into lexical networks. This is consistent with the CLS account of word-learning (Davis and Gaskell, 2009).

While some evidence for consolidation effects was found in Experiment 1, the clearest evidence was in Experiment 3 that provided no German words in the Stroop task. Stroop effects prior to a consolidation period were observed only when the German color words were present in the Stroop blocks (Experiments 1 and 2). Given that novel and German words are paired during learning, the German words in the Stroop test provide contextual information from the learning phase. This context seems critical for the emergence of immediate Stroop effects. It is yet unknown how such contextual cues from learning may facilitate access to the recently learned associations. We suggest three possibilities of contextual support: First, the German Stroop trials may provide a general reminder of the learning situation as a whole and thus facilitate episodic retrieval (cf. Cairney et al., 2011). Second, they may help activating the general semantic field of color, which in turn may facilitate access to the specific meanings. Third, they may provide the specific opportunity to re-process the critical stimuli by which the novel words had been linked to the semantic concepts, thereby facilitating a reactivation of the crucial links (cf. Tamminen et al., 2012). Taking into account that immediate effects of newly learned words are observed in semantically rich learning situations (Mestres-Missé et al., 2007; Freundlieb et al., 2012) and in our first two experiments, the latter explanation, with a retrieval of the specific memory traces including semantic cues seems to be an explanation that fits all of the observed results. Clearly, these alternative explanations for an interaction between memory consolidation and learning context cannot be differentiated on the basis of the current data, and thus should be targeted in future studies.

**Figure 6:**
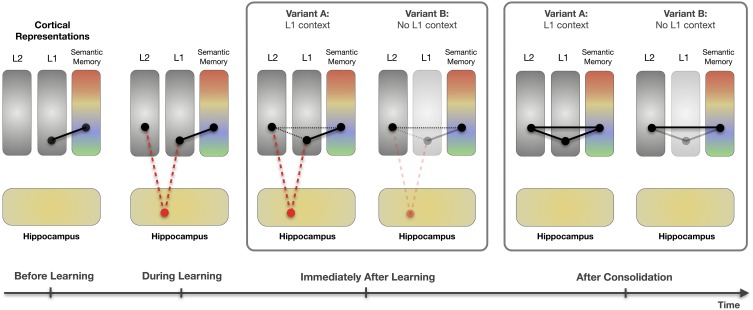
**Memory consolidation of novel color words learned via lexical association**. Before learning, L1 color words and their corresponding color concepts are linked via stable, long-established connections. During learning, L1 words and novel L2 words are paired, mediated via hippocampal activation (dashed red lines). Immediately after learning, L2 words are still linked to their corresponding color concepts via the L1 words. If any direct cortical links exist at all, they are still very weak (dotted black lines). Thus, novel color words best activate their corresponding color concepts when the L1 color words are present in the testing context. If the L1 words are present (Variant A: solid L1 box), L2 words activate their color concepts via the hippocampal link. If there is no L1 context (Variant B: light gray L1 box), there is also no priming of the episodic link between the L2 words and their corresponding concepts, and therefore insufficient conceptual activation. After full consolidation, L2 words have stable cortical links to their L1 counterparts, and to their corresponding color concepts. Therefore, regardless of the presence of L1 words, the L2 words automatically co-activate their corresponding color concepts. (Illustration inspired by Frankland and Bontempi, 2005).

Finally, how can our immediate but context-dependent effect be reconciled with what is known about neural correlates of learning and retrieval? Figure 6 illustrates how learning context may moderate effects of memory consolidation in semantic word learning. We assume that the employed training regime results in an immediate hippocampal association between the German (L1) word and its novel counterpart. This novel association means that the L1 word provides a mediating link in memory between the novel (L2) word and the color semantics. So, even prior to an opportunity for consolidation (provided by sleep, in our case), Stroop effects can be obtained, as long as the L1 word is present in the Stroop task as a contextual cue that “primes” or temporarily strengthens this indirect association. In fact, there is direct evidence for the involvement of the hippocampus during associative learning of the type implemented here: Breitenstein et al. (2005) used event-related fMRI while participants learned novel words in the scanner. Correlated amplitude changes between the hippocampus and neocortical regions were observed, in line with the overall evidence for the importance of the hippocampus in the formation of arbitrary associations in memory (e.g., McClelland et al., 1995; Davachi and Wagner, 2002; Kesner, 2013).

After a period suitable for consolidation, a qualitatively different memory trace seems to be involved in the Stroop effect. There is no longer any need for contextual “priming” from the L1 words. Instead, the novel words operate just as would be expected for words from an established second language, showing clear Stroop effects independent of the L1 context. Potentially, a stronger direct link has now emerged between the new word and the semantics of the word, which means that contextual priming is no longer necessary for swift and obligatory access to the meaning. This is coherent with a systems-consolidation account of the new word memory in which sleep-associated consolidation reduces the dependence on hippocampal mediation and increases the strength of a direct neocortical link between the new word and its meaning (McClelland et al., 1995; Davis and Gaskell, 2009; Takashima et al., 2009). This 24-h change may just be the start of the process, but may still be sufficient to allow context-independent Stroop effects to emerge. Given that semantic access for the L2 words is independent of the L1 words already 24 h after learning, our results stand in contrast to models that assume a prolonged dependence of L2 words on L1 mediation for semantic access (e.g., Kroll and Stewart, 1994).

Putting these results together, the data suggest that although some markers of automaticity in the perception of words are evident soon after learning, the access to meaning becomes more automatic after an opportunity for consolidation (see also Coutanche and Thompson-Schill, 2014; Takashima et al., 2014). Moors and De Houwer (2006) discuss the notion of automaticity with reference to a set of overlapping features. Automatic processes will tend to be ones that are unintentional, uncontrollable, goal independent, autonomous, stimulus-driven, unconscious, efficient and fast. However, these properties may not all co-occur, and it is feasible to think of automaticity as a graded phenomenon. Such a characterization fits well with the current results. Soon after learning, the new words can be processed in a way that is partly automatic. As long as there is sufficient contextual priming, then the new meaning of the novel words is unintentionally and uncontrollably accessed, leading to inhibition of the desired response (indicating the ink color of the word). However, after consolidation there is no longer a contextual requirement, and the meaning of the novel word can be thought of as accessible independently or autonomously (and possibly more efficiently).

These results are also in line with another study that looked at the effects of consolidation on markers of automaticity. Tham, Lindsay, and Gaskell (submitted) used two different effects that have been given as evidence of automaticity: the semantic distance effect and the semantic congruity effect. The authors found that newly learned words would show some hallmarks of automatic processing a few minutes after learning (particularly the semantic distance effect), but that sleep, and particularly slow wave sleep and spindle activity, were associated with the emergence of the semantic congruity effect, which is thought to be a sterner test of automaticity.

In sum, our results stress that careful experimental manipulations are necessary to fully capture the intricate learning and memory processes involved in the acquisition of novel meaningful words. The brain recruits multiple resources to immediately associate newly learned material with well-established knowledge. The context in which learning takes place, and the particular aspects that the learning situation provides or focuses upon, are important for the immediacy of effects that indicate the integration of newly learned words. A stable and strong integration in existing semantic networks, diagnosed by automatic effects in suitable tasks, seems to require consolidation, to become less dependent on contextual cues from the learning situation.

### Conflict of interest statement

The authors declare that the research was conducted in the absence of any commercial or financial relationships that could be construed as a potential conflict of interest.

## Footnotes

^1^There is in fact a lively debate about whether word reading in the Stroop task is in itself fully automatic or whether it can be blocked under extreme experimental circumstances (probably yes; e.g., Besner, 2001). However, the relevant questions here are whether word reading can be avoided through the participant's intention alone (probably not; see, e.g., Experiment 7, Brown et al., 2002), and whether once a word has been read, a participant can avoid to process its semantic content (most probably not; e.g., Marcel, 1983; Dehaene et al., 1998; see also Augustinova and Ferrand, 2014).
