# Supplementary material for: Stroop effects from newly learned color words: effects of memory consolidation and episodic context
Source: Front Psychol. 2015 Mar 12;6:278. doi: 10.3389/fpsyg.2015.00278 (PMC4357220; doi:10.3389/fpsyg.2015.00278)
Supplement: Supplementary file 1 [file DataSheet1.zip › Learning Curves Exp 1 to 3.PDF]

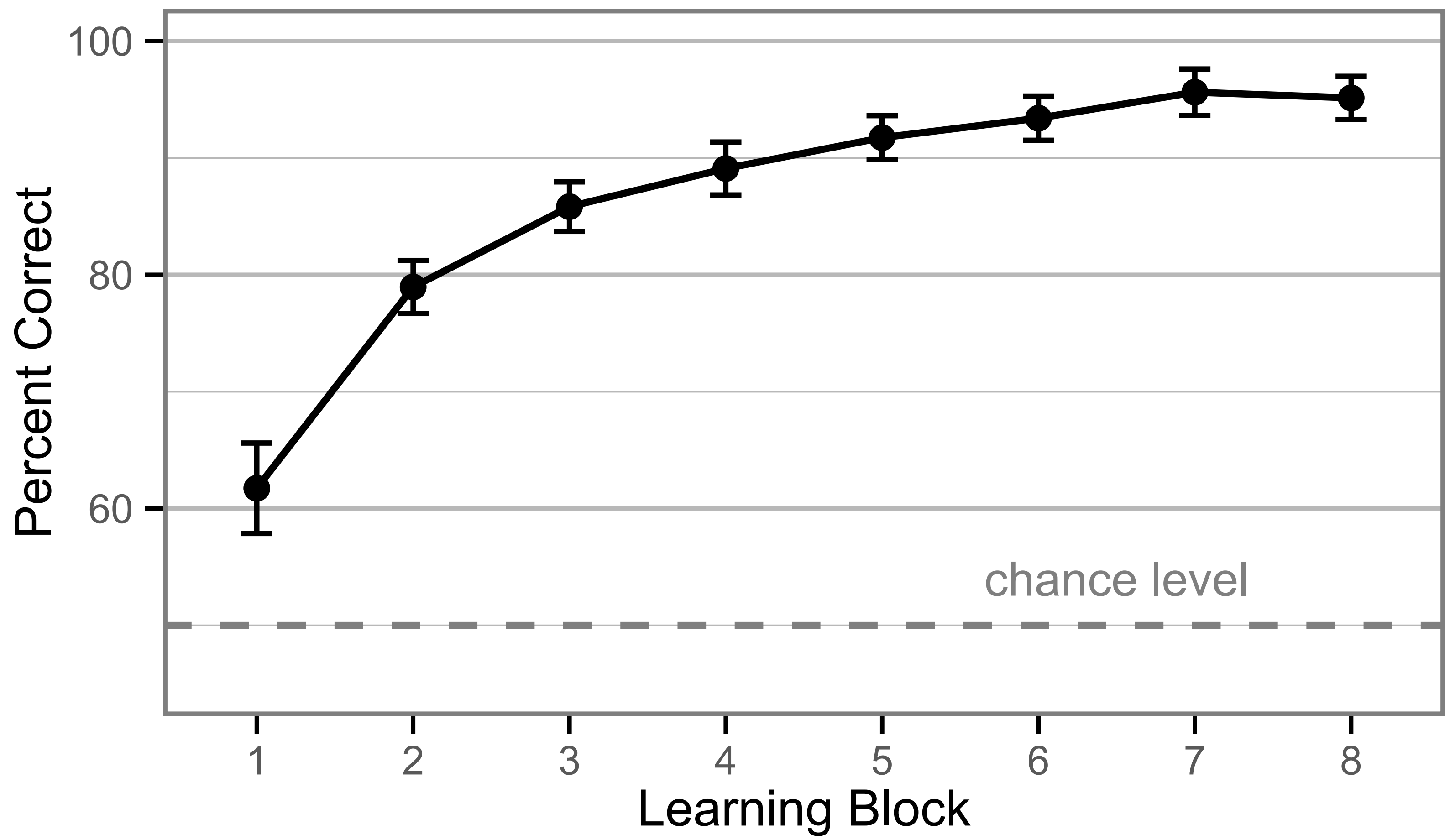

Figure S1. Mean proportion of correct decisions during the learning phase of Experiment 1. On average, a block contained three match and three mismatch trials per novel word.

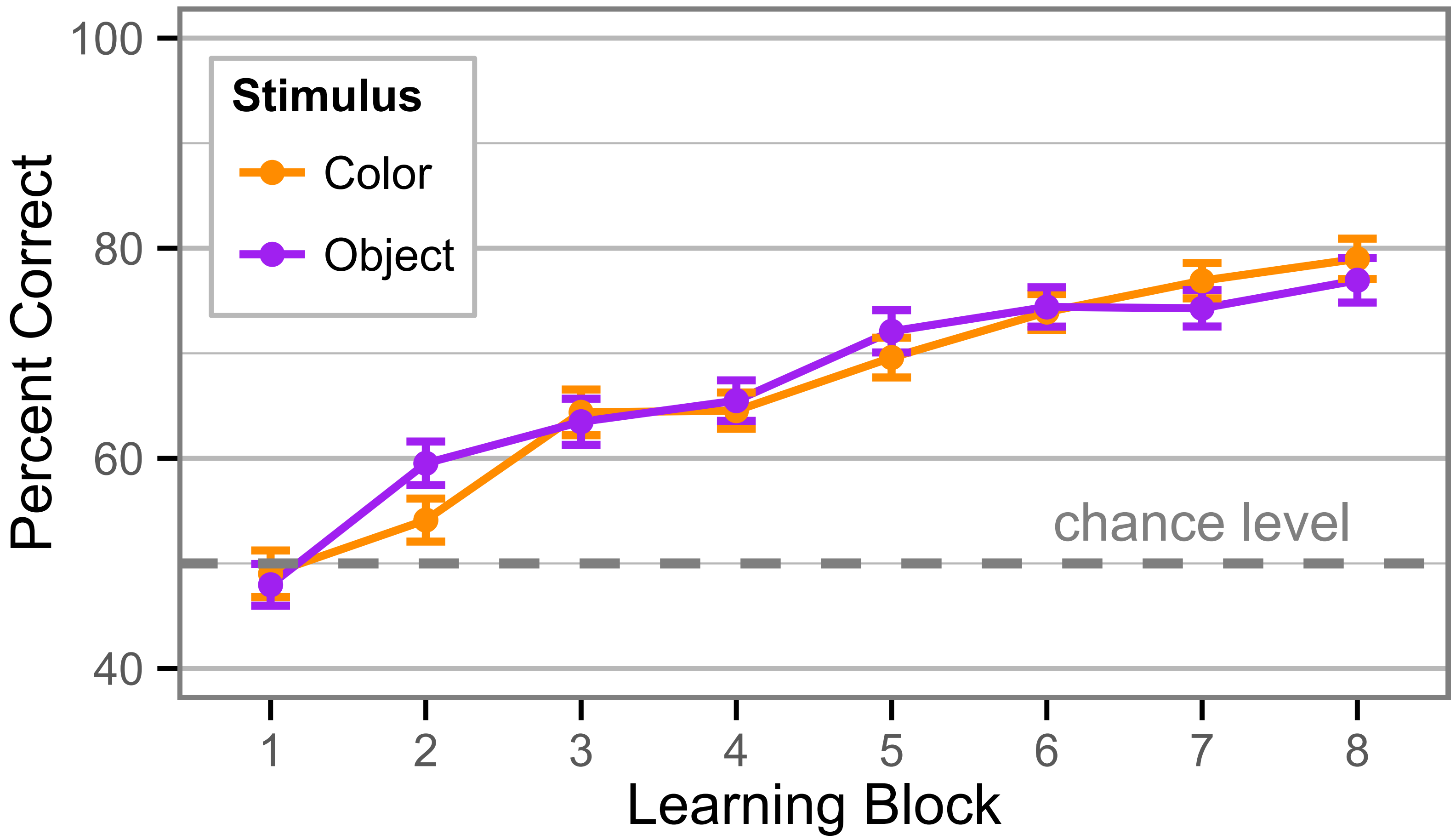

Figure S2. Mean proportions of correct decisions during the learning phase of Experiment 2. Please note: On average, a block contained one match and one mismatch trial per novel word, a third per block compared to Experiment 1.

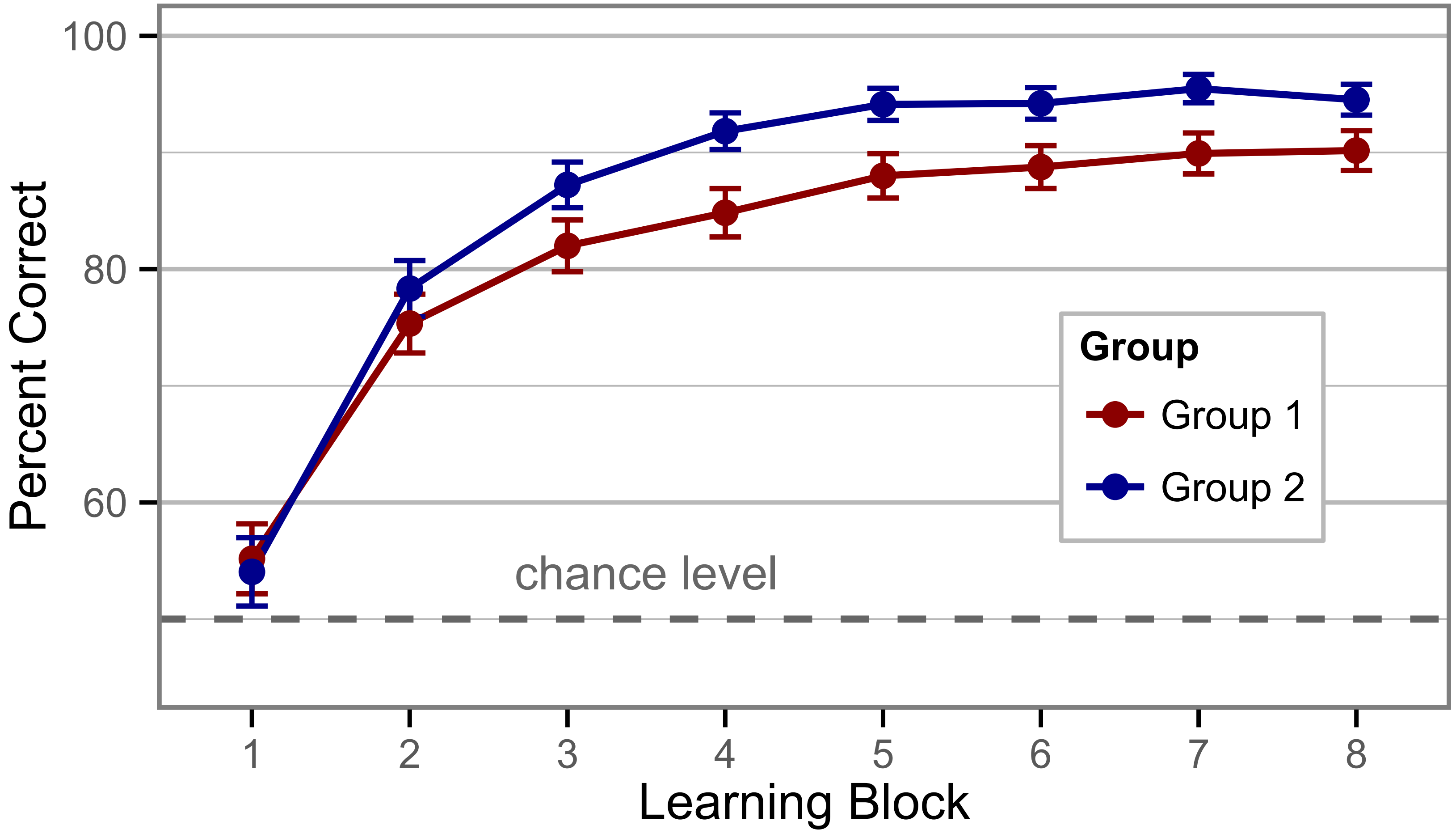

Figure S3. Mean proportions of correct decisions during the learning phase of Experiment 3. On average, a block contained three match and three mismatch trials per novel word, just as in Experiment 1. Note that the error bars shown are within-participant standard errors and thus are not conclusive to visually assess the effect of *Group*.
