# Supplementary material for: Stroop effects from newly learned color words: effects of memory consolidation and episodic context
Source: Front Psychol. 2015 Mar 12;6:278. doi: 10.3389/fpsyg.2015.00278 (PMC4357220; doi:10.3389/fpsyg.2015.00278)
Supplement: Supplementary file 1 [file DataSheet1.zip › Word Assignments Exp 1 and 3.DOCX]

Table S1. The three versions of novel word to German word assignments used in Experiments 1 and 3.

| German | rot | gelb | lila | braun | grün | blau | pink | orange | schwarz | weiß |
| --- | --- | --- | --- | --- | --- | --- | --- | --- | --- | --- |
| *(English* | *red* | *yellow* | *violet* | *brown* | *green* | *blue* | *pink* | *orange* | *black* | *white)* |
| Novel A | gike | dufa | ekir | dapi | tespo | alep | eftu | siba | fupo | rukri |
| Novel B | dapi | tespo | fupo | rukri | siba | gike | alep | ekir | dufa | eftu |
| Novel C | eftu | alep | rukri | fupo | dapi | siba | ekir | gike | tespo | dufa |
