# Supplementary material for: Stroop effects from newly learned color words: effects of memory consolidation and episodic context
Source: Front Psychol. 2015 Mar 12;6:278. doi: 10.3389/fpsyg.2015.00278 (PMC4357220; doi:10.3389/fpsyg.2015.00278)
Supplement: Supplementary file 1 [file DataSheet1.zip › Word Assignments Exp 2.DOCX]

Table S2. The four versions of novel word to German word assignments and the non-lexical items used in Experiment 2.

| Color words |  |  |  |  |  |  |  |  |  |
| --- | --- | --- | --- | --- | --- | --- | --- | --- | --- |
| German | rot | gelb | lila | braun | grün | blau | pink | orange |  |
| *(English* | *red* | *yellow* | *violet* | *brown* | *green* | *blue* | *pink* | *orange)* |  |
| Novel A | gike | dufa | ekir | dapi | alep | eftu | siba | fupo |  |
| Novel B | alep | eftu | fupo | siba | dufa | gike | ekir | dapi |  |
| Novel C | aruh | zopa | okas | feso | iruf | kedo | mupa | uzir |  |
| Novel D | iruf | kedo | mupa | uzir | aruh | zopa | okas | feso |  |
|  |  |  |  |  |  |  |  |  |  |
| Object names |  |  |  |  |  |  |  |  |  |
| German | Mappe | Schachtel | Eimer | Mütze | Sofa | Blüte | Schere | Lappen |  |
| *(English* | *folder* | *box* | *bucket* | *cap* | *sofa* | *blossom* | *scissors* | *cloth)* |  |
| Novel A | aruh | zopa | okas | feso | iruf | kedo | mupa | uzir |  |
| Novel B | iruf | kedo | siba | uzir | aruh | zopa | okas | feso |  |
| Novel C | dapi | ekir | dufa | gike | fupo | siba | alep | eftu |  |
| Novel D | fupo | siba | eftu | alep | dapi | ekir | dufa | gike |  |
|  |  |  |  |  |  |  |  |  |  |
| Non-lexical items | xXx | XxXx | xXXxx | XxXx | XxxXXx | XxxX | XXxxX | XxXXx |  |
